# Standardising Gender and Sex Data Collection in Clinical Care and Research

**DOI:** 10.5694/mja2.70177

**Published:** 2026-03-31

**Authors:** Sav Zwickl, Ada S. Cheung

**Affiliations:** ^1^ University of Melbourne Melbourne Victoria Australia

**Keywords:** gender identity, medical records, population characteristics, research design

To the Editor,

We commend the *Medical Journal of Australia* for its Special Issue on Gender and Health, which highlights persistent sex disparities in conditions, such as myocardial infarction and anxiety disorders and advances an evidence‐based, person‐centred approach to care for trans and gender diverse (trans) people [1]. The article by Graham in this Special Issue of the *MJA* clearly emphasises broader systemic limitations in electronic medical records, demographic data collection and health research design that obscure the influence of sex and gender on health outcomes [2].

Accurate and consistent demographic data are critical to identifying health needs and understanding how gender affects many conditions across the population [3]. Conflating sex and gender, or collecting only one of these variables, masks important differences between cisgender and trans people, who comprise around 0.9% of Australians aged over 16 years [4]. Gender‐affirming hormone therapy may alter risk profiles for conditions with sex‐linked patterns, such as cardiovascular, autoimmune, oncologic or neuropsychiatric conditions [5, 6, 7], but without identifying trans people in research or clinical datasets, such effects cannot be studied.

We recommend that all human research and electronic medical records implement a two‐step approach to gender and sex data collection (Figure 1)—as outlined in the Australian Bureau of Statistics (ABS) Standard [3]—asking:

- ‘How do you describe your gender?’—gender refers to current gender, which may be different to sex recorded at birth and may be different to what is indicated on legal documents, with response options of man or male, woman or female, non‐binary, a different term (please specify), and prefer not to answer; and
- ‘What was your sex recorded at birth?’—with response options of male, female and another term (please specify).

**FIGURE 1:**
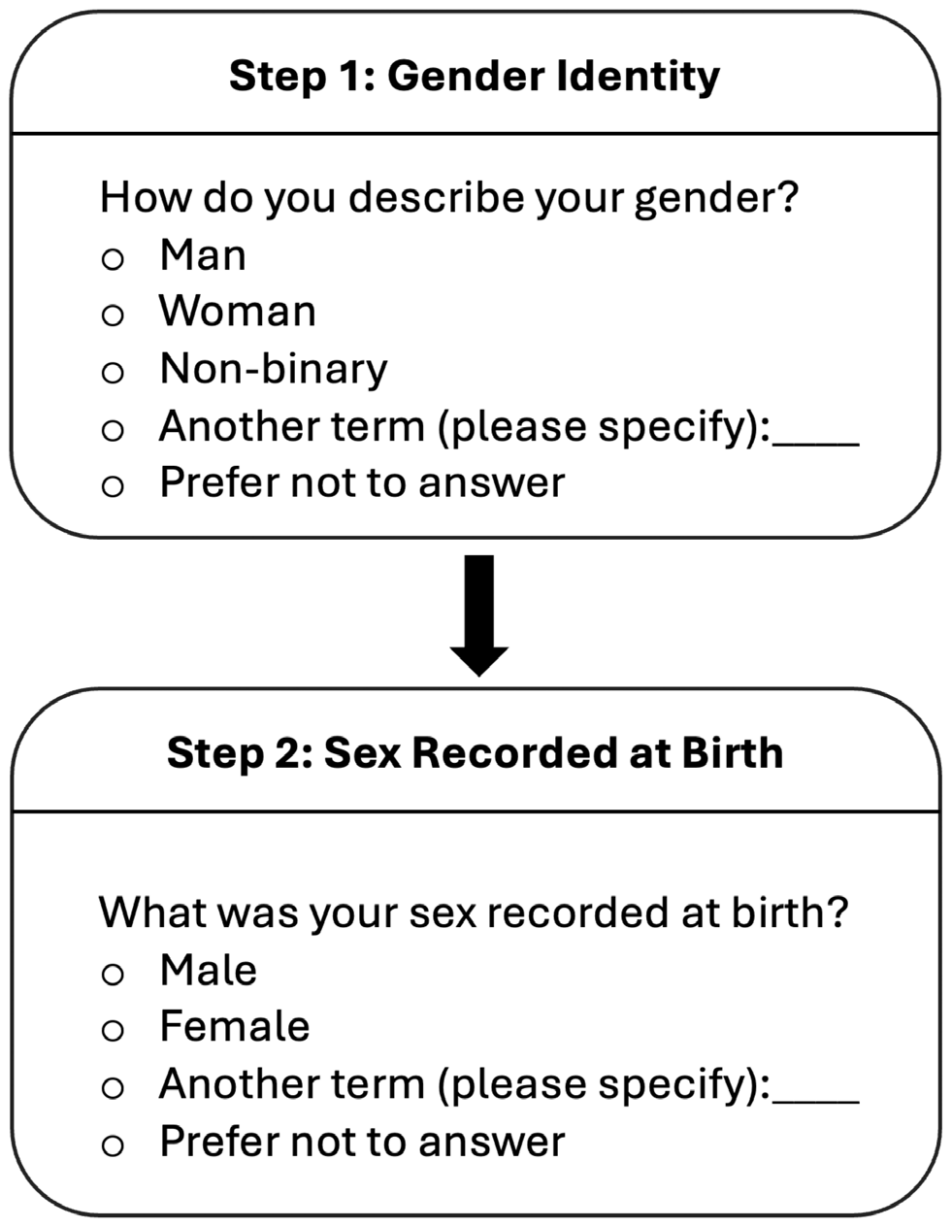
Two‐step method for collecting data on gender and sex. Figure adapted with permission from www.transhub.org.au/allies/researchers, an initiative of ACON NSW, and the Australian Bureau of Statistics [3]. This approach allows determination of whether respondents are cisgender or transgender (trans), including trans men, trans women and non‐binary people recorded female or male at birth.

This approach improves accuracy, respondent acceptability and cross‐system data linkage [8]. Implementing this low‐burden, high‐impact change would enable meaningful inclusion of trans people in health research, strengthen the evidence base on the influence of sex and gender on health, enhance the scientific integrity of Australian data and inform targeted interventions to advance health equity.

National research policy now aligns with this approach: from 2026, National Health and Medical Research Council and Medical Research Future Fund applicants must demonstrate how sex and gender are considered and collected, ideally using the ABS 2020 Standard [3]. Embedding these requirements reflects the joint *Statement on Sex, Gender, Variations of Sex Characteristics and Sexual Orientation in Health and Medical Research* and aims to strengthen the quality, reproducibility and equity of funded research [9]. As current definitions of sex and gender reflect contemporary evidence and are shaped by social concepts, standardisation should be iterative and responsive to change.

Standardising gender and sex data collection is an urgent and achievable step towards realising the vision of evidence‐based, inclusive healthcare articulated throughout this Special Issue of the *MJA*.

## Author Contributions

**Sav Zwickl:** conceptualisation, writing – review and editing. **Ada S. Cheung:** conceptualisation, writing – original draft. Both authors reviewed and approved the final version of the manuscript.

## Funding

Sav Zwickl was supported by a University of Melbourne Faculty of Medicine, Dentistry and Health Sciences Research Fellowship. Ada S. Cheung was supported by a National Health and Medical Research Council Investigator (Grant no. 2008956). Sav Zwickl and Ada S. Cheung were supported by a University of Melbourne Faculty of Medicine, Dentistry and Health Sciences Diversity and Inclusion Grant to develop *Including Trans People in Research: A Practical Guide to Collecting Data on Gender and Sex* (https://www.transresearch.org.au/professional/including). The funders had no role in design, interpretation or publication of this letter.

## Disclosure

Provenance: Not commissioned; externally peer reviewed.

## Conflicts of Interest

The authors declare no conflicts of interest.

## Data Availability

The authors have nothing to report.
